# Genome-Wide Discovery of DNA Polymorphisms in Mei (*Prunus mume* Sieb. et Zucc.), an Ornamental Woody Plant, with Contrasting Tree Architecture and their Functional Relevance for Weeping Trait

**DOI:** 10.1007/s11105-016-1000-4

**Published:** 2016-08-08

**Authors:** Jie Zhang, Kai Zhao, Dan Hou, Junhuo Cai, Qixiang Zhang, Tangren Cheng, Huitang Pan, Weiru Yang

**Affiliations:** Beijing Key Laboratory of Ornamental Plants Germplasm Innovation & Molecular Breeding, National Engineering Research Center for Floriculture, Beijing Laboratory of Urban and Rural Ecological Environment and College of Landscape Architecture, Beijing Forestry University, Beijing, 100083 China

**Keywords:** *Prunus mume*, Weeping trait, Single nucleotide polymorphisms, Insertions/deletions, Gene function

## Abstract

Next-generation sequencing technologies provide opportunities to ascertain the genetic basis of phenotypic differences, even in the closely related cultivars via detection of large amount of DNA polymorphisms. In this study, we performed whole-genome re-sequencing of two mei cultivars with contrasting tree architecture. 75.87 million 100 bp pair-end reads were generated, with 92 % coverage of the genome. Re-sequencing data of two former upright mei cultivars were applied for detecting DNA polymorphisms, since we were more interested in variations conferring weeping trait. Applying stringent parameters, 157,317 mutual single nucleotide polymorphisms (SNPs) and 15,064 mutual insertions-deletions (InDels) were detected and found unevenly distributed within and among the mei chromosomes, which lead to the discovery of 220 high-density, 463 low-density SNP regions together with 80 high-density InDel regions. Additionally, 322 large-effect SNPs and 433 large-effect InDels were detected, and 10.09 % of the SNPs were observed in coding regions. 5.25 % SNPs in coding regions resulted in non-synonymous changes. Ninety SNPs were chosen randomly for validation using high-resolution melt analysis. 93.3 % of the candidate SNPs contained the predicted SNPs. Pfam analysis was further conducted to better understand SNP effects on gene functions. DNA polymorphisms of two known QTL loci conferring weeping trait and their functional effect were also analyzed thoroughly. This study highlights promising functional markers for molecular breeding and a whole-genome genetic basis of weeping trait in mei.

**Electronic supplementary material:**

The online version of this article (doi:10.1007/s11105-016-1000-4) contains supplementary material, which is available to authorized users.

## Introduction

Mei (*Prunus mume* Sieb. et Zucc., 2n = 2x = 16) is a popular ornamental plant widely cultivated in East Asia. Being an important member of the genus *Prunus* and a species domesticated for over 3000 years, it plays a pivotal role in phylogenic studies of the Rosaceae. Besides, it owns many highlighted ornamental features, such as colorful corollas, pleasant fragrance, and weeping trait (Chen 1996; Zhang et al. 2015). Weeping trees, characterized by soft and pendant branches, are used worldwide as landscape plants due to their aesthetic value in our living environment. Because of specific structure design, weeping trees are believed to play a pivotal role in understanding the genetic mechanism of plant architecture and have drawn increasing attention during the past decades (Barthélémy and Caraglio 2007; Petersen and Krost 2013; Yamanouchi et al. 2009). However, the underlying genetic and molecular principles for weeping trait remain an unresolved mystery. Developing weeping cultivar with higher ornamental value is still a laborious job. Thus, dissecting the genetic basis of weeping trait is an urgent step for effective breeding of weeping cultivars, especially the woody ornamental plants. The development of a genome-level resource for mei will assist the exploration and improvement of genetic studies of this important ornamental plant, and will provide a better perceptive of weeping trait in *Prunus*.

Genome-wide polymorphisms identification is crucial to study the genetic basis of phenotypic differences within species (Causse et al. 2013; Srivastava et al. 2014), since they are major determinants of phenotypic variations, and their interaction with the environment is vital for the expression of a trait (Subbaiyan et al. 2012). With the release of the mei genome and the development of next-generation sequencing (NGS) techniques (Zhang et al. 2012), it is now much more feasible to learn genome-wide genetic variation through large-scale re-sequencing of whole genomes in a cost-effective way (Bentley 2006). The detection of a massive number of DNA polymorphisms, such as single nucleotide polymorphisms (SNPs) and insertions-deletions (InDels), is one of the most important applications of NGS techniques (Huang et al. 2013; Varshney et al. 2009). SNPs have gained importance over other DNA markers because of their inherent advantage of high abundance, high-throughput capability, and cost-effectiveness (Henry and Edwards 2009). In addition to massive number of SNPs, InDels have also become valuable DNA markers being applied for QTL mapping and marker-assisted selection with characteristics of much cheaper charge, relatively simple genotyping, and easy transferability between populations (Hayashi et al. 2006; Pan et al. 2008). SNPs and InDels have already been wildly employed in breeding programs for marker-assisted selection, quantitative trait locus mapping, and association mapping (Ma et al. 2014; McCouch et al. 2010; Ren et al. 2010; Zou et al. 2014). Besides, the distributions of SNPs and InDels within a genome also affect the expression and function of genes. Among these genetic variants, non-synonymous and large-effect DNA polymorphisms are particularly important as they are predicted to alter protein function. Therefore, detecting polymorphisms related to functional changes of genes is critical for investigating the reasons for phenotypic differences (Jain et al. 2014). Moreover, the statistic of annotation results of genome-wide genes containing variants can explain the genetic basis of weeping trait to some extent.

With this background, the present study was carried out with the aim of discovering genome-wide identical DNA polymorphisms in a weeping cultivar of mei compared with three mei upright cultivars, with the reference genome of mei using as a “bridge.” Whole-genome re-sequencing of two mei cultivars were carried out using the Illumina Hiseq 2000 platform. The sequence reads generated were then mapped to the high-quality reference genome sequences of mei, and mutual genome-wide variations were uncovered through comprehensive detection of SNPs and InDels across the genomes among different tree architectures. Furthermore, DNA polymorphisms in QTLs conferring weeping trait were detected and annotated. The discovery and annotation of the genetic variations in this study will provide vital clues that will help to unravel the genetic basis of weeping trait and provide promising functional markers in mei.

## Materials and Methods

### Plant Materials

The cultivars used for re-sequencing were the upright cultivar “Liu Ban” of mei (2000-36) from QingDao, China (36.20169° N, 120.4162° E) and the weeping cultivar “Fen Tai ChuiZhi” of mei (2001-40) from WuHan, China (30.54526° N, 114.39511° E). These were selected based on their contrasting phenotypic divergence in tree architecture (Supplementary Fig. 1).

### Sequencing and Reads Mapping

Genomic DNA was extracted from young leaves of “Fen Tai ChuiZhi” and “Liu Ban” cultivars in vigorous growth period using the DNA Secure plant kit (TianGen, Beijing, China). The quality and quantity of the DNA was checked by agarose gels and Bioanalyzer 2100 (Agilent Technologies, Singapore). Following quality assessment, genomic DNA was randomly fragmented using sonification (Covaris M220) and was then purified by DNeasy Plant Mini Kit (Qiagen, Hilden, Germany). Terminal repair was conducted for the digested DNA fragments using T4 DNA polymerase and Klenow DNA polymerase (NEB), together with the phosphorylation modification for the 5’ end. A single nucleotide (A) overhang was added subsequently using Klenow Fragment (NEB) at 37 °C. Solexa adapters were ligated to the A-tailed fragments, and then sticked to flow cell to perform bridge polymerase chain reaction (PCR). DNA fragments of the desired length (400–600 bp) were then gel purified and PCR was conducted again to increase the template amount. Then, DNA quantity of the templates were determined by Invitrogen Qubit fluorometer (Life Technologies, Carlsbad, USA), and bridge amplication was conducted on the surface of the flow cell chip-set. After further amplification in the cluster station, the flow cell was moved to the Illumina HiSeq 2000 system (Illumina, Inc; San Diego, CA, USA) and pair-end sequencing was performed according to the manufacturer’s recommendations.

Raw data were filtered using SOAPfilter software to generate high-quality reads by the following criteria (Jain et al. 2014). First, low-quality reads and reads containing adaptor contamination were removed. Second, reads were discarded once the low-quality bases accounted for more than 20 % of the total reads. Third, reads with more than 5 % unknown bases (N) were also discarded. Besides, repetitive reads caused by PCR amplification were removed. Only reads that were at least 35 bp after passing Illumina’s filter were retained. These high-quality reads were then aligned to the mei reference genome (http://prunusmumegenome.bjfu.edu.cn) using Burrows-Wheeler Aligner (v 0.7.10-r789) algorithm under the default parameters (Li and Durbin 2009b). Duplication-reads were filtered using picard-tools, and those mapping to unique location of the reference genome were retained.

### Identification and Validation of SNPs and InDels

Since the two cultivars “Liu Ban” and “Fen Tai ChuiZhi” differ in a number of ornamental traits, data of former resequenced upright cultivars (“Kouzi Yudie” (1999-31) and “Fen Ban” (2000-15)) were applied to assist detection of DNA polymorphisms closely related to weeping trait (Sun et al. 2013). Raw re-sequencing data of the former upright cultivars “Kouzi Yudie” and “Fen Ban” were downloaded from the NCBI database and then filtered as mentioned above. For the detection of DNA polymorphisms, the reference genome of mei was used as a “bridge” to sequentially detect variants between cultivars. The pair-end reads of these cultivars were aligned to the mei reference genome by BWA software. After that, the aligned reads dataset of “Fen Tai ChuiZhi” was compared with the three aligned reads dataset of upright cultivars separately. Only those identical DNA polymorphisms between the “Fen Tai ChuiZhi” genome with the other three upright cultivars were deemed as variants more associated with weeping trait.

Base quality scores recalibration and local realignment were performed using the Genome Analysis Toolkit (GATK) to get analysis-ready sequence alignment files (Jain et al. 2014; Zou et al. 2014). The obtained files were then processed and adapted for the SNP and InDel calling program using the Unified Genotyper module of GATK. Polymorphisms were called with the standard call confidence and standard emit confidence both set to 30.0, while the minimum base quality scores set to 10e. After that, raw variants were filtered out using recalibrate variant quality scores (VQSR) in GATK to reduce the false discovery rate of SNPs and InDels. In short, the variants calling results of SAMTOOL and GATK software were intersected to produce a reliable calling set which served as reference for subsequent analysis. Three parameters (total depth of coverage (DP), the rank sum test for mapping qualities (MQRankSum), and the rank sum test for the distance from the end of the reads (ReadPosRankSum)) were calculated according to the data set. The SNP/InDel recalibration model was built based on these three parameters, and VQSLOD value for each SNP/InDel was calculated on the basis of the recalibration model. To increase the specificity of variant calling, threshold of tranche level was set to 90, while the VQSLOD ≥1.8 for SNP calling set and VQSLOD ≥1.5 for InDel calling set. The filtered variants were then applied for further analyses. To investigate the density of these DNA polymorphisms, their frequency was calculated in each 100-kb interval with a 1-Mb sliding window across the whole genome. The distribution of SNPs and InDels markers on each chromosome was visualized using Circos (Krzywinski et al. 2009).

In addition to “Liu Ban,” “Fen Tai ChuiZhi,” “Kouzi Yudie,” and “Fen Ban,” nine weeping cultivars and seven upright cultivars of mei were selected for SNP validation (Supplementary Table 1). Ninety putative SNPs were randomly selected and screened across these mei cultivars applying high-resolution melt (HRM) analysis (Herrmann et al. 2006). HRM primers were designed using Beacon Designer 7.91 (Supplementary Table 2). After that, HRM PCRs were conducted on a Bio-Rad CFX96 qRT-PCR machine and HRM data were analyzed by Precision Melt Analysis software according to its manual.

### Genome-Wide SNPs and InDels Annotation

To assess the function of the variants in genomic regions, a SnpEff predictor database file in binary format (.bin) was created to locate each SNP within annotated transcripts or intronic regions using SnpEff software (ver 3.1h) (Cingolani et al. 2012). The “Prunus mume v1.0 genome” and annotation GFF files were used as references (Zhang et al. 2012). Default parameters of the SnpEff software were implied to generate the predictor database and to perform the variant effect analysis of the DNA polymorphisms in annotated transcripts within the 5000 bases of the upstream and downstream areas of the Open Reading Frames. The position of the variants on the genome, the transitions/tranversions ratio (Ts/Tv), the gene ID, the gene name, warnings, and the effect of the variants were available in the output file (Cingolani et al. 2012). PFAM domains in the proteins were predicted by searching against the PFAM database (release 27.0) using the HMMscan program (ver 3.0), with the threshold of BLAST set to 10^−5^ (Jain et al. 2014).

### Identification of DNA Polymorphisms in Weeping Trait-Related QTL Regions

QTL regions for weeping trait were identified according to former association mapping analysis (unpublished data) and linkage analysis of our research group (Zhang et al. 2015). The genes present in the QTL region were detected according to the mei genome annotation GFF files. Effects of variants within the QTL regions were predicted using the SnpEff software as described above.

Genes in the QTL regions were then screened for polymorphism at the amino acid level, and the genes showing at least one change at the amino acid level were considered candidates. For a more detailed functional annotation, eukaryotic orthologous group (KOG) class was assigned by searching query gene sequence against KOG database available at NCBI. In addition, GO enrichment analyses were further conducted for these candidate genes.

## Results

### Reads Mapping and Coverage of the mei Reference Genome

Whole-genome sequencing of “Liu Ban” and “Fen Tai ChuiZhi”, yielded 75.87 million 100-bp paired-end reads, which comprised 6.78 Gb of high-quality raw data. A total of 67,792,732 high-quality reads were obtained from the two cultivars after appropriate quality filtering as described in the “Materials and Methods” part. The number of high-quality reads varied from 30.97 to 36.82 million for the two cultivars (Table 1). 91.57 and 91.26 % of the obtained reads were successfully mapped onto the mei reference genome separately by applying the BWA software (Li and Durbin 2009a), which covered 92 % of the total genome on average. Base quality of the reads mapping to the genome were also detected. Unique mapped reads with minimum mapping quality of 30, accounting for 63.31 and 62.84 % of the reference genome, respectively, were used for downstream analysis.

**Table 1:** Summary of sequence data and mapping statistics of “Liu Ban” and “Fen Tai ChuiZhi” cultivar

	“Liu Ban”	“FenTai ChuiZhi”
Total reads (Mb)	34.40	41.47
High-quality reads (Mb)	30.97	36.82
Sequencing depth	12.15	14.45
Total reads mapped (%)	91.57	91.26
Genome coverage (%)	92.2	93.45
Unique reads mapped (%)	82.23	81.51
Genome coverage (%)	87.16	88.5
Unique reads mapped with MAPQ30	63.31	62.84
Genome coverage (100 %)	80.42	80.41

*MAPQ30* mapping quality of 30

### Detection and Distribution of SNPs and InDels

Identical variations detected in “FenTaiChuiZhi” cultivar compared with the three upright cultivars of mei were considered as potential weeping-related variations. In total, 172,381 polymorphisms were detected. The most common variants were SNPs, while InDels were present in similar numbers and proportions; these variants represent in approximately 8.74 % of the total DNA polymorphisms (Table 2). The detailed information for the SNPs and InDels along with flanking sequences and predicted primers is shown in Supplementary Table 3 and Supplementary Table 4.

**Table 2:** Polymorphism detected in “Fen Tai ChuiZhi” compared with the three upright cultivars of mei

Chromosome no.	No. of SNP	SNP/100 kb	No. of InDels	InDels/100 kb	No. of Insertions	No. of Deletions
Pm1	22966	85.84	2138	7.99	944	1194
Pm2	34164	81.17	3289	7.81	1470	1819
Pm3	17104	70.22	1603	6.58	761	842
Pm 4	18929	79.08	1798	7.51	802	996
Pm 5	18904	72.32	1760	6.73	811	949
Pm 6	18211	85.53	1758	8.26	806	952
Pm 7	13939	81.78	1362	7.99	667	695
Pm 8	13100	75.94	1356	7.86	626	730
Total	157317	79.11	15064	7.58	6887	8177

*SNP* single nucleotide polymorphisms

**Fig. 1:**
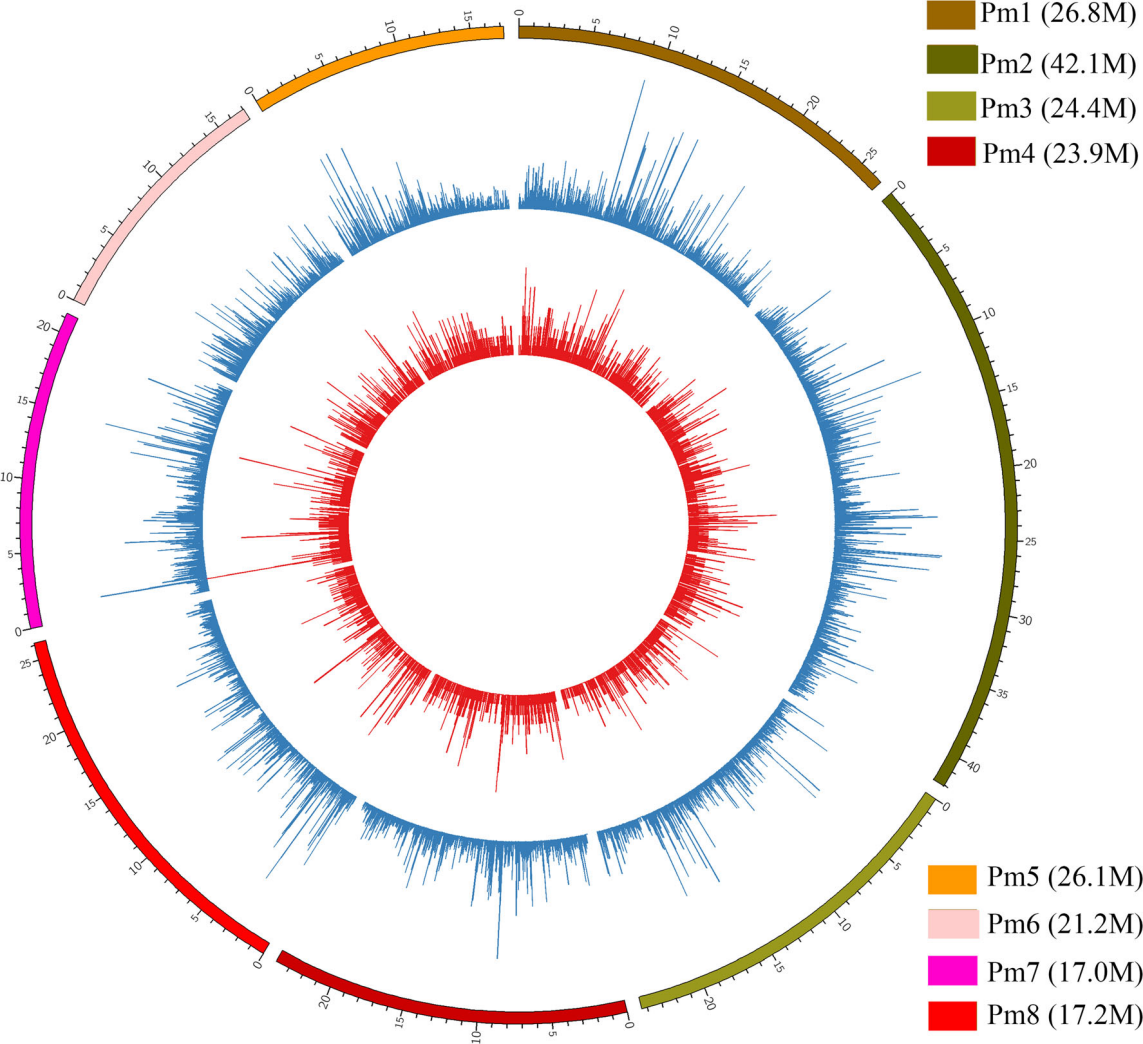
Distribution of mutual SNPs and InDels in “Fen Tai ChuiZhi” compared with the three upright cultivars of mei separately in the chromosomes. All tracks are plotted in 100 Kb windows. Length of each chromosome (Pm) of mei was shown in *bracket*. The *y*-axis ranges from 0 to 100 %. **a** SNP density was shown in *blue*. **b** InDel density was shown in *red*

Distribution of the variants among the eight chromosomes of mei was detected. The total number of SNPs and InDels which were found to be directly proportional to the chromosome lengths varied across chromosomes (Fig. 1). The largest number of variants was detected in chromosome 2 and the lowest was in chromosome 8. In total, the average densities (numbers per 100 kb) of detected polymorphisms between weeping and upright cultivars were 79.11 (SNPs) and 7.58 (InDels). The highest SNP density was ascertained in chromosome 1 (85.84/100 kb) and the lowest in chromosome 3 (70.22/100 kb). On the other hand, InDel density was the highest in chromosome 6 (8.26/100 kb) and the lowest in chromosome 3 (6.58/100 kb) (Table 2).

Also, we observed that SNPs and InDels were not uniformly distributed within the eight chromosomes (Fig. 1). With a 1-kb sliding window, the number of variants within the 100 kb region across the mei genome was calculated. Two hundred twenty high-density SNP regions of 100 kb each, with ≥500 SNPs were detected, and chromosome 6 owns the maximum high-density SNP regions. Four hundred sixty-three low-density SNP regions of 100 kb each, with <1 SNP were found across the whole genome. Likewise, the distribution of InDels within the chromosomes was not uniform (Fig. 1). Only 80 high-density regions of 100 kb each with >50 InDels were identified, and the maximum high-density InDel regions were also observed in chromosome 6.

### Characteristics and Validation of SNPs and InDels

The SNPs detected were classified as transitions (A/G and T/C; Ts) or transversions (A/T, A/C, T/G, C/G; Tv) based on nucleotide substitutions. In general, the frequency of transitions was slightly lower than transversions, and the ratio between Ts/Tv was 0.84 (Supplementary Fig. 2a). Among the transitions, the frequency of A/G transitions was significantly higher than T/C transitions. As for transversions, the frequency of C/G transversions was much elevated than the others.

Out of the total 6887 insertions and 8177 deletions, the variation in the length of the insertions was ranged from 1 to 63 bp, while the length of deletions was up to 116 bp (Supplementary Fig. 2b). Most of the InDels (70.13 %) were single nucleotide or di-nucleotides insertions and deletions. Only one deletion was longer than 100 bp and all insertions were smaller than 100 bp. The corresponding amount of the InDels was inversely proportional to the length of the InDels, which has also been detected in former researches (Arai-Kichise et al. 2011).

To validate the quality of the identified SNPs, 90 SNPs were randomly chosen for experimental validation. The locations of SNPs selected in each chromosome were presented in Supplementary Fig. 3. Of the 90 SNPs, 84 (93.3 %) contained the predicted SNPs, which is a fairly high validation rate. SNPs tested negative are presented by red lines in Supplementary Fig. 3. Meanwhile, 69 (77 %) SNPs showed polymorphism between weeping type and upright type of mei according to the sequences of the 20 mei cultivars; the detailed information are presented in Supplementary Table 2.

### Genome-Wide DNA Polymorphisms Annotation

SNPs are with small differences but have great impact on the variation of genomes and the phenotypic traits (Zheng et al. 2011). Supplementary Table 5 shows the exact account of SNPs in each effect class. Ninety percent SNPs were classified as modifiers, since they located outside the gene. Among the modifiers, 47.72 % were in downstream regions of a gene, while 31.85 % SNPs were in intergenic regions and 10.3 % were in intronic regions. Moderate SNPs which changed the amino acid sequence accounted only for 5.25 %, and the fraction of low-effect variants was 4.67 %. Among the SNPs detected in coding sequences, 52.91 % were non-synonymous coding SNPs and 46.98 % were synonymous ones. Meanwhile, the large-effect SNPs which modify splice sites, stop or start codons represented the smallest class, with only 322 SNPs (0.21 %). In addition to the above SNPs, InDels can also cause large effects on gene functions. Annotation of InDels indicated that 28.24 % of the InDels were observed in 4253 genes in “Fen Tai ChuiZhi” compared to upright cultivars. 55.58 % InDels were detected in downstream regions.

**Fig. 2:**
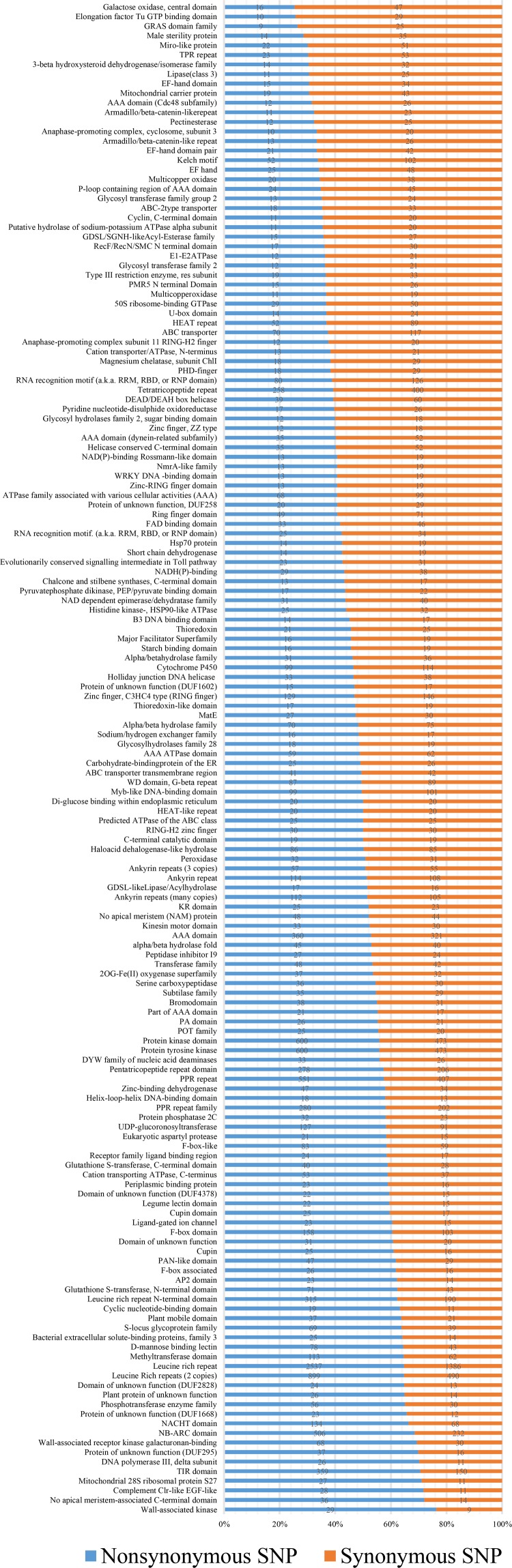
Numbers and distribution of non-synonymous and synonymous SNPs in different Pfam families of mutual SNPs in “Fen Tai ChuiZhi” compared with the three upright cultivars of mei separately. The Pfam families with 30 or more SNPs were listed, and are arranged according to the percentages of non-synonymous and synonymous SNP. Non-synonymous SNPs are presented in *blue bars*, while synonymous SNPs are presented in *red bars*

To better understand the effect of SNPs on gene function, the distribution of the SNPs in proteins containing Pfam conserved domains was further analyzed in detail. Predicted genes containing SNPs could be assigned to 4489 types of Pfam domains (Supplementary Table 6). About 16.71 % SNPs were found in leucine-rich repeat (8.49 %), pentatricopeptide (PPR) repeat family (3.26 %), and protein kinase domain (4.96 %) which function as an “on” or “off” switch in many cellular functions (Schmitz-Linneweber and Small 2008; Xu et al. 2014). Meanwhile, about 10 % SNPs were related to MYB transcription factor or domain and needed further attention. We then paid attention to SNPs occurred in genic regions, which seem to be more important for functional analyses (Lestari et al. 2014). Figure 2 shows the number of SNPs detected in each Pfam group. About 39.1 % SNPs were detected in genes encoding leucine-rich repeat (PF12799.2, PF13855.1), pentatricopeptide repeat (PPR) domain (PF01535.15, PF12854.2), protein tyrosine kinase, and protein kinase (PF00069.20, PF07714.12). 23.4 % SNPs were found in the leucine-rich repeats domain which contained the most abundant SNPs, with 6.1 % SNPs in the PPR domain, and 9.40 % SNPs were associated with protein tyrosine kinase and protein kinase. Meanwhile, all the sequences encoding the above domains owned higher ratios of non-synonymous to synonymous SNPs (ns/s SNPs) than average. In addition, the ratio of ns/s SNPs in each Pfam group was also counted. Among these, the sequences encoding Wall-associated kinase (PF08488.6) and no apical meristem-associated C-terminal domain (PF14303.1) had the highest ns/s SNPs ratios, and elongation factor Tu GTP binding domain and galactose oxidase, central domain had the lowest ratios.

Pfam analysis was further conducted for genes containing large-effect SNPs which were predicted to have a potentially disabling effect on gene function. Among these large-effect SNPs, 203 SNPs were expected to induce premature stop codons, 20 to alter initiation methionine residues, 66 to disrupt splicing donor or acceptor sites, and an additional 33 to remove the annotated stop codons, resulting in longer open reading frames. The majority of the large-effect SNPs were enriched in leucine-rich repeats domain (PF13855.1, PF13504.1, PF00560.28, PF12799.2), PPR repeat domain (PF13041.1, PF12854.2, PF13812.1), protein kinase domain (PF00069.20, PF07714.12), and Ankyrin repeats (PF12796.2, PF13857.1, PF00023.25, PF13606.1), most of which were consistent to the above Pfam analysis results.

### Candidate Genes and DNA Polymorphisms Underlying Weeping Trait-Related QTLs

The region of two candidate QTLs conferring weeping trait ranged from 8.36 to 11.69 M in chromosome 7 of mei (Zhang et al. 2015). Applying the annotation project files of mei, 359 genes were finally detected in the QTL regions. DNA polymorphisms of these genes were analyzed to further dissect the potential weeping trait-related variants and candidate genes. In total, 2292 SNPs and 339 InDels were detected in the QTL regions. 51.4 % SNPs were predicted in the downstream area, and 29.93 % were in the intergenic region, while only 10.16 % SNPs were detected in coding sequences. The distribution of InDels was detected which was quite similar to that of SNPs, with more than half of the InDels (59.59 %) were detected in the downstream region (Supplementary Table 7).

**Fig. 3:**
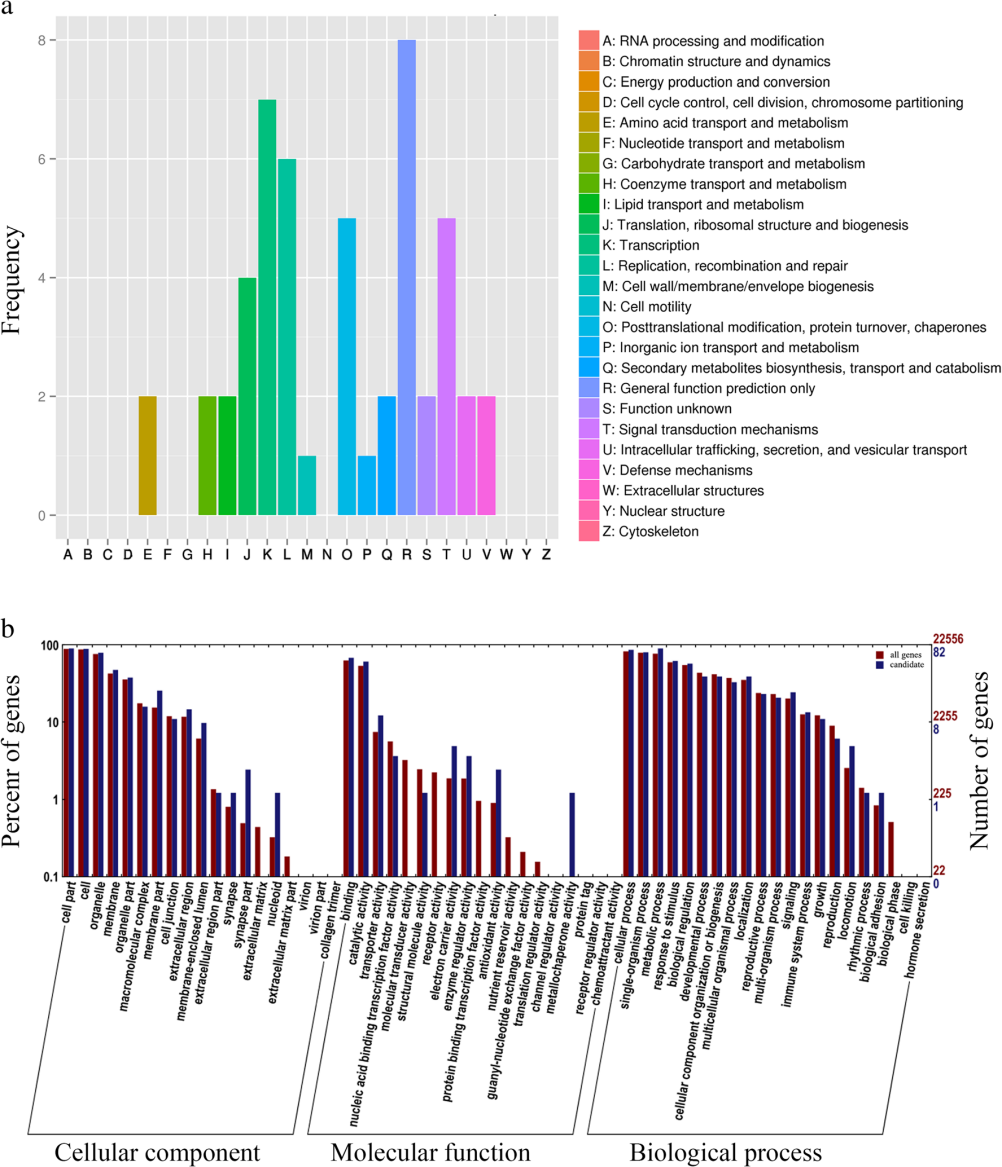
Functional categorization of candidate genes with amino acid changes identified within the QTL regions of weeping trait. **a** Distribution of the KOG classes in candidate genes with amino acid changes within the QTL regions. **b** GO enrichment analysis of the candidate genes with amino acid changes within the QTL regions

Subsequently, we laid more emphasis on variants which have an effect on amino acids, since these variants are more important for functional analysis. Two hundred thirty-nine SNPs together with seven InDels were predicted to cause amino acid changes of 118 genes. Eukaryotic orthologous group (KOG) analysis was performed to investigate the putative functions of these candidate genes. Excluding genes of general functions prediction, genes involved in transcription followed by replication, recombination, and repair were most represented. Besides, the genes related to posttranslational modification, protein turnover, chaperones, and signal transduction mechanisms were also abundant (Fig. 3a). Gene ontology enrichment analysis corroborated the results of KOG analysis. The genes responsive to stimulus and involved in signaling were significantly represented in the candidates. Besides, genes involved in binding activity, transporter activity, electron carrier, and localization were also significantly enriched in the GO terms (Fig. 3b).

## Discussion

### Whole-Genome Re-Sequencing for Detecting Genome-Wide DNA Polymorphisms

With the rapid development of next-generation sequencing technologies, it is now much more reliable to discover DNA polymorphisms on a genome-wide scale, which plays a vital role in unraveling the genetic basis of phenotypic differences at genomic level. By re-sequencing a weeping cultivar and an upright cultivar of mei, together with application of former re-sequencing data of two upright mei cultivars, we uncovered 172,381 SNPs and InDels which were expected to be more related with tree architecture. This is a first report on the genome-wide detection of genetic variation for tree architecture, which will be of great value for further genotype-phenotype studies and for molecular breeding of this important ornamental and economical trait.

In this study, only 8 % of reads could not be mapped onto the reference genome of mei, indicating a low percentage of repeated sequences in its genome. In addition, the average genome coverage reached 13.3× (Table 1), which ensured a high level of confidence in the detected DNA polymorphisms. The average polymorphisms (SNPs and InDels) rate was 86.69 per 100 kb, offering relatively high-density coverage across the entire mei genome. Moreover, 90 SNPs were chosen for validation applying HRM analysis. 93.3 % of the randomly selected SNPs contained the predicted SNPs, and the validation rate was slightly higher than the 92.5 % obtained in soybean and 92.9 % in common bean (Hyten et al. 2010; Zou et al. 2014), which indicated the high quality of SNPs identified. The polymorphisms of the 90 SNPs were further detected in 10 weeping cultivars and 10 upright cultivars of mei, and 77 % SNPs were supposed to be related to weeping trait of mei. These SNPs are predicted to be promising markers linked to weeping trait and could be applied in early selection of weeping trait.

### Distribution of DNA Polymorphisms in Mei Genome

Analyses of the numbers of mutual SNPs and InDels in “Fen Tai ChuiZhi” compared with the three upright cultivars of mei indicated that the numbers of polymorphisms in each chromosome was proportional to their physical sizes. The distribution of DNA polymorphisms was found to be uneven across and within the mei chromosomes, which was in line with former researches in mei and other species (Fresnedo-Ramírez et al. 2013; Sun et al. 2013). Further analysis of SNPs and InDels in each chromosome revealed high-density SNP/InDel regions and low-density SNP/InDel regions along the chromosomes. The occurrence of such regional variations in the genome had also been reported in mei as well as in rice and wheat (Somers et al. 2006; Subbaiyan et al. 2012; Sun et al. 2013). More intensive SNP distribution was observed in chromosome 7 (Fig. 1), which was consistent with former linkage analysis showing that the major locus controlling weeping trait was located on chromosome 7 of mei (Zhang et al. 2015). As a whole, the high-density SNP/InDel regions especially those in chromosome 7 may contain promising candidate genes and functional markers pertaining to weeping trait. This suggests that genome-wide DNA polymorphisms detection can be an effective tool to dissect the genetic mechanism of a specific trait.

### Analysis of Functional Polymorphisms Associated with Weeping Trait

Only 10.09 % of the polymorphisms occurred in coding regions based on the annotation of the SNPs across the whole genome. Three hundred twenty-two specific large-effect SNPs and 8252 non-synonymous coding SNPs are likely candidate markers, as they may change the integrity of gene functions. The results of the Pfam domain analysis of genes containing SNPs and large-effect SNPs were really conspicuous to some extent; leucine-rich repeat, PPR repeat family, and protein kinase domain were most abundant, suggesting that the difference between tree architecture may be associated with the difference in protein-protein interactions. Among the Pfam domains containing genes of interest is the PPR repeat family which is candidate of weeping trait in soybean (Kong 2012). Thus, SNPs in sequences, encoding PPR repeat family, need further verification in mei. Ten percent SNPs concerning to MYB transcription factor may be possible functional markers of weeping trait. This is because weeping trait has been reported to be controlled by a regulatory gene causing differential expression of the downstream network (Sugano et al. 2004).

SNPs occurring in genic regions are more important for functional analyses, and we further paid attention on SNPs in coding regions. Pfam analysis of these SNPs also indicated that the most abundant SNPs were in sequences encoding leucine-rich repeat, PPR domain, protein tyrosine kinase, and protein kinase. Combined with former Pfam analyses of natural and large-effect SNPs, we supposed that weeping trait is tightly linked to protein interactions and RNA metabolism after gravity perception. On the other hand, SNPs detected in wall-associated kinase (PF08488.6) and no apical meristem-associated C-terminal domain (PF14303.1), with the highest ns/s SNPs ratios, were supposed to be promising functional markers associated with weeping trait. The reason is that the structure of the cell wall in different tree architectures varied from each other (Andersson Gunnerås 2005; Coutand et al. 2004).

The availability of the annotated reference genome allowed us to indentify candidate genes and alleles underlying the QTLs conferring weeping trait detected by our research group. The 2292 SNPs and 339 InDels detected in the QTL regions can be used to narrow down the candidate regions by linkage and association analysis in the future. Besides, the KOG and GO analysis result of the 118 candidate genes with amino acid changes accorded with former researches of the weeping trait mechanism, that weeping trait has a differential expression in genes response to gravity (Dong 2014.5; Zhang 2008).

In short, the present study has detected 172,381 SNPs and InDels totally. Among them, 15,575 SNPs and 4253 InDels were detected in genic regions. Pfam analysis for natural SNPs, large-effect and genic SNPs suggested that genes containing leucine-rich repeat, PPR repeat family, MYB transcription factor domains own the most abundant variants, which indicated that weeping trait may be linked to protein interactions and RNA metabolism after gravity perception, and these SNPs were supposed to be functional markers for weeping trait. DNA polymorphisms detected in the QTL region especially the variants which caused the amino acid change can be applied in further linkage analysis to narrow down the scope of weeping candidate genes. Hence, the SNPs and InDels detected in this study contribute to comprehensive understanding of current limited molecular mechanism of weeping trait and are promising functional markers of weeping trait.

## Electronic Supplementary Material

Below is the link to the electronic supplementary material.

Supplementary Fig. 1 Phenotypes of weeping trait and upright trait of the two mei cultivars for re-sequencing. (DOC 33 kb)

Supplementary Fig. 2 Characteristics of mutual SNPs and InDels in ‘Fen Tai ChuiZhi’ compared with the three upright cultivars of mei separately. a. Frequency of different substitution types in mutual SNPs detected; b. Distribution of the length of mutual insertions and deletions identified. The x-axis represents the number of nucleotides of insertions (red) and deletions (blue). The y-axis represents the number of deletions and insertions at each length. (TIF 10439 kb)

Supplementary Fig. 3 Location of the 90 SNPs markers selected for validation in each chromosomes of mei. (TIF 431 kb)

Supplementary Table 1 Mei cultivars selected for SNP validation and genotyping by the HRM method. (TIF 374 kb)

Supplementary Table 2 Primers used for HRM analysis and SNP validation. (DOC 42 kb)

Supplementary Table 3 The detailed information of the mutual SNPs in ‘Fen Tai ChuiZhi’ compared with the three upright cultivars of mei separately. (DOC 149 kb)

Supplementary Table 4 The detailed information of the mutual InDels in ‘Fen Tai ChuiZhi’ compared with the three upright cultivars of mei sepatately. (XLSX 52034 kb)

Supplementary Table 5 Distribution of the effects of SNPs in ‘Fen Tai ChuiZhi’ compared with the three upright cultivars. (XLS 10428 kb)

Supplementary Table 6 The List of the Pfam gene families of mutual SNPs in ‘Fen Tai ChuiZhi’ compared with the three upright cultivars of mei separately. (DOC 36 kb)

Supplementary Table 7 Distribution of the SNP and InDels effect in ‘Fen Tai ChuiZhi’ compared with the three upright cultivars of mei in QTLs conferring weeping trait. (XLS 1183 kb)
