# Supplementary material for: Genome-Wide Discovery of DNA Polymorphisms in Mei (Prunus mume Sieb. et Zucc.), an Ornamental Woody Plant, with Contrasting Tree Architecture and their Functional Relevance for Weeping Trait
Source: Plant Mol Biol Report. 2016 Aug 8;35(1):37–46. doi: 10.1007/s11105-016-1000-4 (PMC5306074; doi:10.1007/s11105-016-1000-4)
Supplement: Supplementary file 1 — Phenotypes of weeping trait and upright trait of the two mei cultivars for re-sequencing. (DOC 33 kb) [file 11105_2016_1000_MOESM10_ESM.doc]

**Supplementary Table 7** Distribution of the SNP and InDels effect in ‘Fen Tai ChuiZhi’ compared with the three upright cultivars of mei in QTLs conferring weeping trait.

| SNP Types | No. | InDel Types | No. |
| --- | --- | --- | --- |
| INTRON | 188 | INTRON | 45 |
| DOWNSTREAM | 1178 | DOWNSTREAM | 202 |
| INTERGENIC | 686 | INTERGENIC | 82 |
| SYNONYMOUS_CODING | 108 | STOP_LOST | 1 |
| NON_SYNONYMOUS_CODING | 125 | CODON_INSERTION | 3 |
| STOP_LOST | 1 | CODON_DELETION | 2 |
| STOP_GAINED | 5 | FRAME_SHIFT | 4 |
| Total SNP | 2292 | Total InDel | 339 |
