# Supplementary figures and images for: Genome-Wide Discovery of DNA Polymorphisms in Mei (Prunus mume Sieb. et Zucc.), an Ornamental Woody Plant, with Contrasting Tree Architecture and their Functional Relevance for Weeping Trait
Source: Plant Mol Biol Report. 2016 Aug 8;35(1):37–46. doi: 10.1007/s11105-016-1000-4 (PMC5306074; doi:10.1007/s11105-016-1000-4)

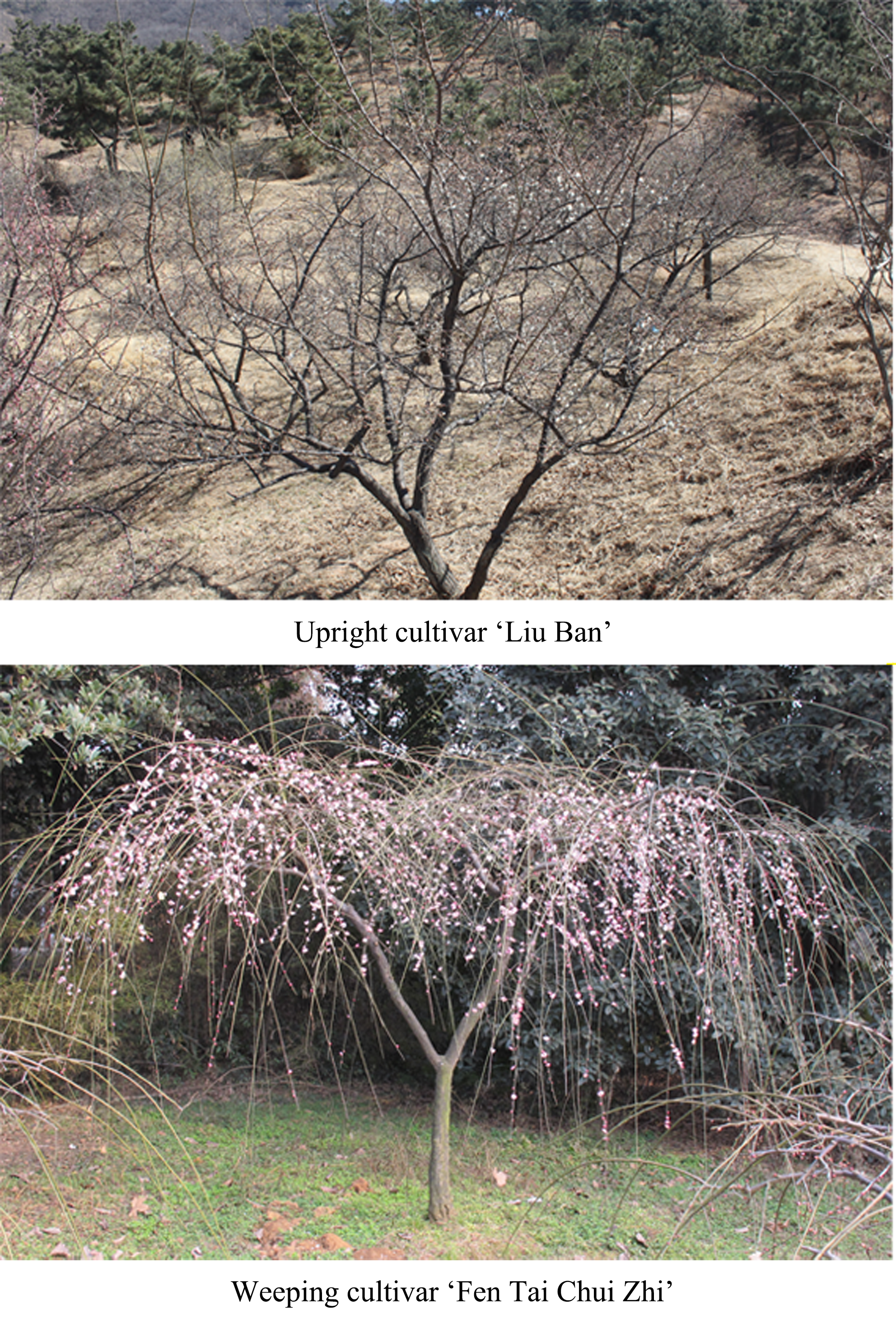

Supplement: Supplementary file 2 — Characteristics of mutual SNPs and InDels in ‘Fen Tai ChuiZhi’ compared with the three upright cultivars of mei separately. a. Frequency of different substitution types in mutual SNPs detected; b. Distribution of the length of mutual insertions and deletions identified. The x-axis represents the number of nucleotides of insertions (red) and deletions (blue). The y-axis represents the number of deletions and insertions at each length. (TIF 10439 kb) [file 11105_2016_1000_MOESM1_ESM.tif]

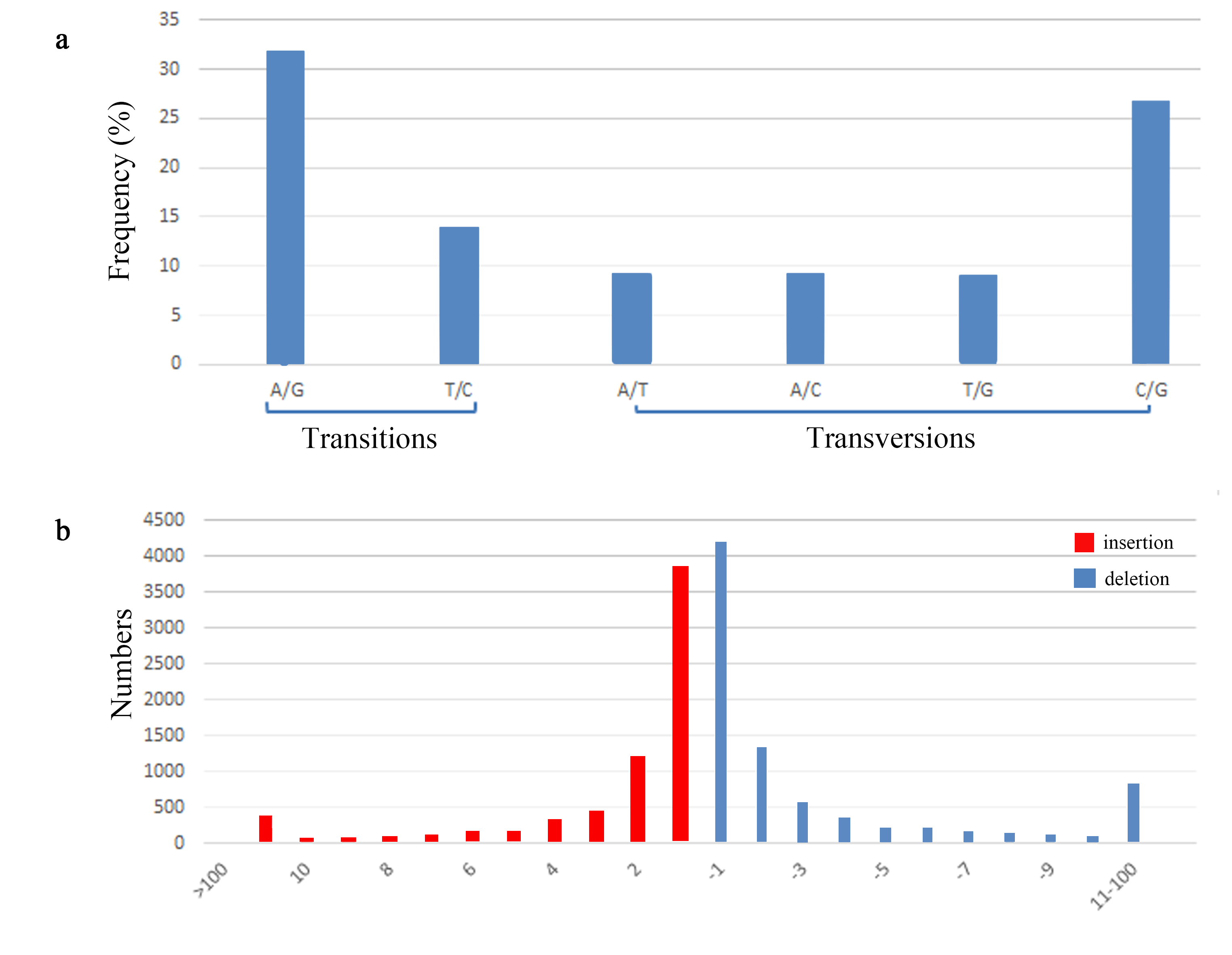

Supplement: Supplementary file 3 — Location of the 90 SNPs markers selected for validation in each chromosomes of mei. (TIF 431 kb) [file 11105_2016_1000_MOESM2_ESM.tif]

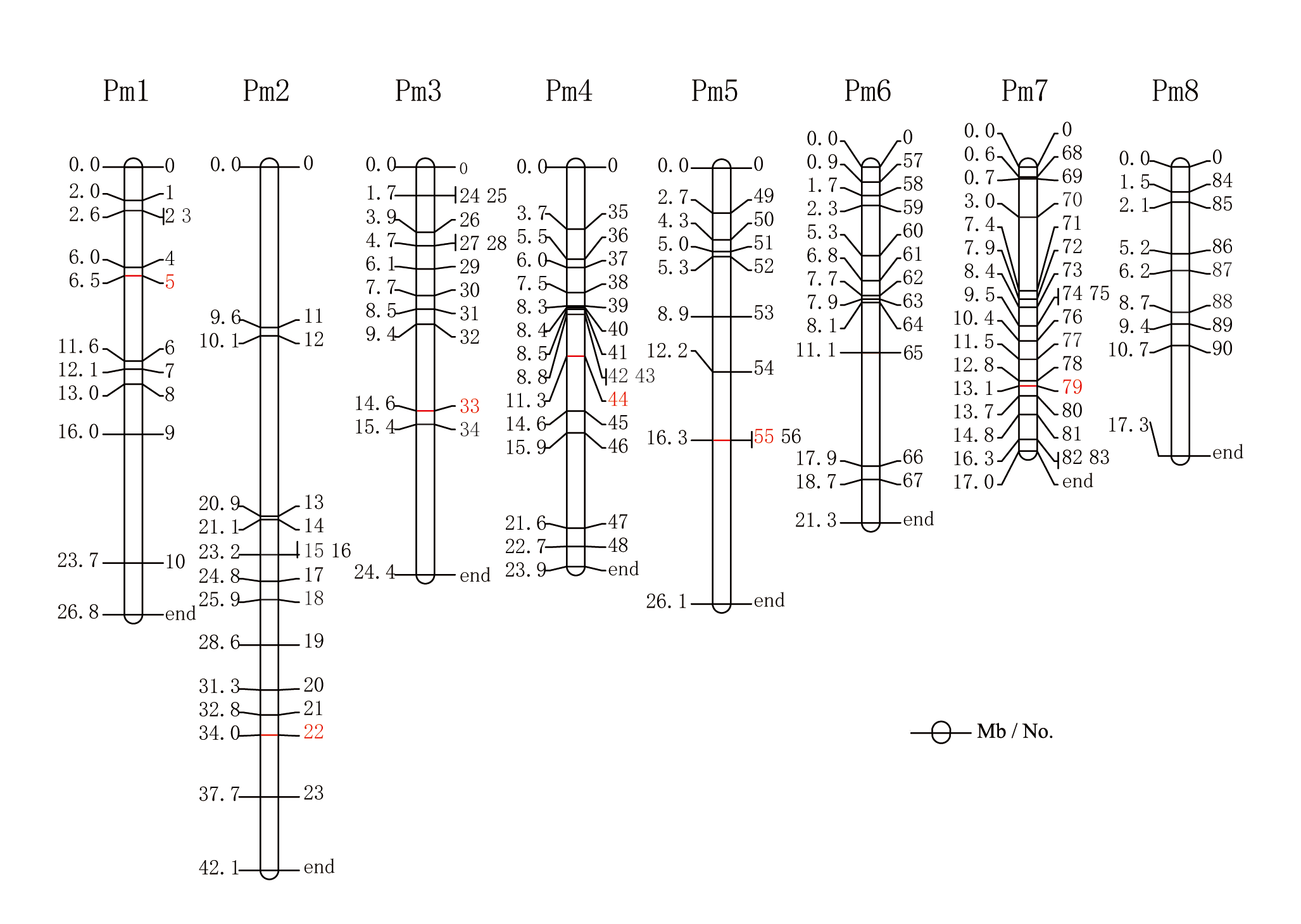

Supplement: Supplementary file 4 — Mei cultivars selected for SNP validation and genotyping by the HRM method. (TIF 374 kb) [file 11105_2016_1000_MOESM3_ESM.tif]
