# Supplementary material for: Genome-Wide Discovery of DNA Polymorphisms in Mei (Prunus mume Sieb. et Zucc.), an Ornamental Woody Plant, with Contrasting Tree Architecture and their Functional Relevance for Weeping Trait
Source: Plant Mol Biol Report. 2016 Aug 8;35(1):37–46. doi: 10.1007/s11105-016-1000-4 (PMC5306074; doi:10.1007/s11105-016-1000-4)
Supplement: Supplementary file 5 — Primers used for HRM analysis and SNP validation. (DOC 42 kb) [file 11105_2016_1000_MOESM4_ESM.doc]

**Supplementary Table 1 Mei cultivars used for SNP validation and genotyping by the HRM method**

| Accession numbers | Cultivar name | Type |
| --- | --- | --- |
| 2000-36 | ‘Liu Ban’ | Upright |
| 1999-31 | ‘Kouzi Yudie’ | Upright |
| 2000-15 | ‘Fen Ban’ | Upright |
| 2001-75 | ‘LianHu Fen’ | Upright |
| 2003-12 | ‘Dong Zhi’ | Upright |
| 2001-110 | ‘Wu Yu Yu’ | Upright |
| 1999-41 | ‘Sanlun Yudie’ | Upright |
| 1999-55 | ‘Xiao’ou Gongfen’ | Upright |
| 1999-37 | ‘Ning Xin’ | Upright |
| 1999-44 | ‘Subai Taige’ | Upright |
| 2001-40 | ‘Fen Tai ChuiZhi’ | Weeping |
| 2001-103 | ‘ShuangBi ChuiZhi’ | Weeping |
| 2001-24 | ‘Danbi Chuizhi’ | Weeping |
| 2001-06 | ‘Can Xue’ | Weeping |
| 2001-25 | ‘Danfen Chuizhi’ | Weeping |
| 1999-17 | ‘Fenpi Chuizhi’ | Weeping |
| 2000-41 | ‘Moshan Chuizhi’ | Weeping |
| 2001-70 | ‘JinHong ChuiZhi’ | Weeping |
| 2001-107 | ‘TiaoXue ChuiZhi’ | Weeping |
| 2005-18 | ‘Fu Fen ChuiZhi’ | Weeping |

HRM: high resolution melt . SNP:single nucleotide polymorphism.

All the information for the cultivars of mei can be checked on <http://www.ishs.org/sci/icralist/20.htm>.
