# Supplementary material for: Genome-Wide Discovery of DNA Polymorphisms in Mei (Prunus mume Sieb. et Zucc.), an Ornamental Woody Plant, with Contrasting Tree Architecture and their Functional Relevance for Weeping Trait
Source: Plant Mol Biol Report. 2016 Aug 8;35(1):37–46. doi: 10.1007/s11105-016-1000-4 (PMC5306074; doi:10.1007/s11105-016-1000-4)
Supplement: Supplementary file 6 — The detailed information of the mutual SNPs in ‘Fen Tai ChuiZhi’ compared with the three upright cultivars of mei separately. (DOC 149 kb) [file 11105_2016_1000_MOESM5_ESM.doc]

**Supplementary Table 5** Distribution of the effects of SNPs in ‘Fen Tai ChuiZhi’ compared with the three upright cultivars.

| Large effect | Total | 322 |
| --- | --- | --- |
|  | Splice site donor | 26 |
|  | Splice site acceptor | 40 |
|  | Stop lost | 33 |
|  | Stop gained | 203 |
|  | Start lost | 20 |
| Moderate effect | Total | 8,252 |
|  | Non synonymous coding | 8,252 |
| Low effect | Total | 7,344 |
|  | Non synonymous start | 3 |
|  | Synonymous stop | 14 |
|  | Synonymous coding | 7,327 |
| Modifier | Total | 141,399 |
|  | Upstream | 13 |
|  | Downstream | 75,077 |
|  | Intergenic | 50,110 |
|  | Intron | 16,199 |
| Total SNPs | 157,317 | |
