# Supplementary material for: Genome-Wide Discovery of DNA Polymorphisms in Mei (Prunus mume Sieb. et Zucc.), an Ornamental Woody Plant, with Contrasting Tree Architecture and their Functional Relevance for Weeping Trait
Source: Plant Mol Biol Report. 2016 Aug 8;35(1):37–46. doi: 10.1007/s11105-016-1000-4 (PMC5306074; doi:10.1007/s11105-016-1000-4)
Supplement: Supplementary file 9 — The List of the Pfam gene families of mutual SNPs in ‘Fen Tai ChuiZhi’ compared with the three upright cultivars of mei separately. (DOC 36 kb) [file 11105_2016_1000_MOESM8_ESM.doc]

**Supplementary Table 2** Primers used for HRM analysis and SNP validation.

| Chromosome ID | SNP Position | Reference Base | Alter Base | Forward Primer (5'-3') | Reverse Primer (5'-3') | SNP |
| --- | --- | --- | --- | --- | --- | --- |
| Pm1 | 12078602 | C | T | ACACACATGAAGCCAATGGA | CCGCCTGTCTACGAACTCAT | Y |
| Pm1 | 15980164 | A | T | GTTGGCCGATTCCTTAGTGA | TTCGGGGACACAAGAAAAAG | Y |
| Pm1 | 13010465 | A | G | AAGATGTGGCACTCAAAGGG | ATTTCGTGGAGCAGGTTTTG | Y |
| Pm1 | 11604428 | A | G | GATCTCCTTGCATGGCATTT | ACTGGTTTATCCGTTGGCAG | Y |
| Pm1 | 6477359 | G | T | TGGGGACCCATCAAAATTAG | TTGGTTTTCCTCTTACGTTGG | N |
| Pm1 | 2035028 | T | C | TGTTCTTTGATGAAGGCCAA | TCCCTTGGATTGGTGAATGT | Y |
| Pm1 | 6009167 | C | T | TTCCAATGGACTGTTTGTCG | GCTTTGCTTTGCAATGATGA | N |
| Pm1 | 23701643 | C | G | TGGGTTCGGTTCTTCTTTCTT | GACTTTGAGGCCCAATACCA | Y |
| Pm1 | 2643974 | A | C | AATGATTATCAAACGGGCCA | GGTTGGCATGCTAAGTTTTTG | Y |
| Pm1 | 2639526 | T | C | TTGCTTTTTGCTGTCTCCCT | GGACCCATTGAGAGCCAATA | Y |
| Pm2 | 23204614 | A | G | AATTTGGACCACCAGAGAGG | CACAGCAGGACAAGAACCAG | N |
| Pm2 | 20937712 | C | T | CTTTTGTTCATAAGCGTCCG | TGGCCAATGTTTCTTCAACA | Y |
| Pm2 | 24841264 | A | G | AGCTCTTTGGACGACGCTAA | AAAGGAGGCCCAGATTCATT | Y |
| Pm2 | 33994762 | A | G | AACTAAGGCCATGTTTGGCA | CATGTTTCAGGCTCGCTGTA | N |
| Pm2 | 10060172 | A | G | ACCTATTTGCCTTGCCTCCT | TTGATCAAGCCCATCAGAAA | Y |
| Pm2 | 28631722 | A | G | TTGGATGATGCACGACTCTC | TGTTGCAGATCCCACAAACA | Y |
| Pm2 | 23178417 | G | C | ACCACATCCAAACATTGCCT | GAGCAATGGCAATGGAGTTT | N |
| Pm2 | 31308873 | A | T | CTGCAGAAAATCCTTGGGAA | CTCGCGTCAAGTGTTGAGAA | Y |
| Pm2 | 9640401 | A | G | AGCATGCAAACACGATGAAG | TCGTTTTGCCCTTACTGGTT | Y |
| Pm2 | 21067728 | C | G | TGGTCTTCAGACTGTCGCAC | GATCCGTAGTCCTCCGTGAA | N |
| Pm2 | 37696022 | C | G | AACACATATCTCAAAAGTGGCCT | AGGTTCCAATGAAGTGCCAG | N |
| Pm2 | 25880142 | A | G | CCCATGTTGGTGCTCTTTTT | CACCACACCAGTGTCTGAGG | Y |
| Pm2 | 32779277 | T | C | AGCTTCGATTTCCTTGCAGA | TTCAAGTCTACCTCGGCGAT | Y |
| Pm3 | 7718134 | A | G | TTCCTATTATGGCAGGCAGG | TGCCACAGTCATGGCTCTAT | Y |
| Pm3 | 3875558 | T | C | TTCTCTTCCCCAT  CACGTTC | AATGGCTTGGTTCAAAGTCG | Y |
| Pm3 | 14622574 | T | C | GCAGCAGATGAAAATGGTGA | CTGAATCCTCTCCACCCAAA | N |
| Pm3 | 9426298 | T | G | CCATTTCCAACCATATTGGG | ACTCCTGCAGCTCCATCATC | Y |
| Pm3 | 6059505 | A | G | AGTGGATGCTAC  ACACGCA | CCCGCTTCAGTGATTTCTTT | Y |
| Pm3 | 15383062 | A | G | CGACCCTTCCAACTGATTTC | CCAATTCCATCACCTTCCAC | Y |
| Pm3 | 8503968 | C | T | ACGACCAAACGCAAAGAATC | GTGTCATGCAGACCTGGCTA | Y |
| Pm3 | 1691203 | C | T | AGGTTTAGTCGTGCACTGGG | GGCAATTCCTCAGGTAACCA | Y |
| Pm3 | 4667039 | C | A | TGGCAGAACACTCATCTTGC | ATCGTAGGCCTCGTCAGCTA | N |
| Pm3 | 1693612 | A | T | GTCGAGGCGAATAAGTTTGG | AGCACAACCCCATCTCTCAC | Y |
| Pm3 | 4655727 | T | A | GGATGTATGTGTTGCGTTGC | AGAGCTTTGGCCATCTTCAA | Y |
| Pm4 | 7529859 | C | T | TCTCCGACAGCACATACTCG | GCGACTTGCAAATTCCTTTC | Y |
| Pm4 | 8843954 | A | G | TCAGCCACATTGTTGACGTT | AAGCCAGGAACAGTTGGAAA | Y |
| Pm4 | 8526728 | G | A | GTGGCTTTTGGTATGGCAGT | AAGCCACGTTGATGGAATGT | Y |
| Pm4 | 15944756 | C | A | TTACTTGGTTGGGCATGACA | CCAACTCGTATGGATAAATGGA | N |
| Pm4 | 6018237 | C | G | ACCGACCAGTCCTTCAACTG | TCGATGGCTTCAAATTTCCT | Y |
| Pm4 | 22704080 | A | G | GCCGAACCAAAACCAAGTAA | GCCACTGACACGTCAGACAG | Y |
| Pm4 | 3689945 | G | T | TGAAGAGCGTGAAAGTGGTG | ACCAATGGAGATGCGGATTA | Y |
| Pm4 | 8285362 | A | C | CTGACCGTTGTGTTGAATGC | TCTCAGTGCGCCATTGATAG | Y |
| Pm4 | 8409683 | C | A | TGGGTTCCATGGAGTAAAGG | GTTCATTTGATTGGTGCGTG | Y |
| Pm4 | 11268630 | C | T | TTCTGAAAGGGAGTGGTTGG | TCGAAATCCCTGGTCTCATC | N |
| Pm4 | 14591575 | A | C | CGCCTCTCATTACTACGCAA | AATTATTAGGGGCCCGTTTG | Y |
| Pm4 | 5447872 | G | A | TCGCACCCAAATACCAAGAT | AGGCGTTGTAGACGAGCAAT | Y |
| Pm4 | 8840631 | C | T | AAGGAGCTATGCAACTCCGA | ACCGACACCACATTGACTGA | Y |
| Pm4 | 21592147 | C | T | GATCGGAAATTCTCGTTGGA | ACCTTGGAGACATGCAAAGG | Y |
| Pm5 | 5281629 | C | T | TGGAGACAAATCCCAACACA | ATCTCGCTCAATCCAAATGC | Y |
| Pm5 | 8848794 | G | A | TGAGGTTTCAAGGACTGAAGG | ATTCACAATTCCCTGCTTCG | Y |
| Pm5 | 16254581 | C | T | TTTCCATGGCTCTTTTGGTC | CTTTGGCTGGCTTCATCTTC | N |
| Pm5 | 2710742 | T | A | CGAAATTCCGGAAGAACAAA | GGCTCTTTCCAACACGACAT | Y |
| Pm5 | 5033516 | A | G | AACGGGACCTGGTTCTATGA | CTTCCATACAATCCGGCAGT | Y |
| Pm5 | 16251582 | C | G | TTGTATGCAAGGTTGACGGA | ATTGGTAGACACAGGGCAGC | N |
| Pm5 | 4296544 | G | C | ACCCACAAAAGAACTGTGCC | AGGAGAGCATGGGCTGTAGA | Y |
| Pm5 | 12225947 | C | T | TCGAATTTCCACCAAGAAGAA | ACAGTGGCTTGCAGGAAGTT | Y |
| Pm6 | 8065950 | G | T | TGAGAGATTCTTGTGTGCGG | ACAGACAGACAGACCCCACC | Y |
| Pm6 | 6799285 | A | T | ATTCTTGGTTCCGCCCTTAT | TTCCAGACCTTTGTCCATGA | N |
| Pm6 | 2249804 | A | C | CTCATCTCCACCCCTTTCTG | TGGGAAGGAAGAAGACATGG | Y |
| Pm6 | 17889930 | A | G | CGTCCGTTGCATATCTCAAA | CACCCAGAAAAGTAAGGCCA | Y |
| Pm6 | 11079844 | C | A | ACTGTTCAGCGGACCATTTC | AACCTTTCATCGGTTTGCAT | N |
| Pm6 | 1729892 | C | G | TCTCGTGTCCATCAAGTCCC | ATGGGTTCATGAAGGTGGAA | Y |
| Pm6 | 18662549 | T | C | GGAAGAACAGGTGCAGAAGC | TTTTGCAGCTGAGCAACATC | Y |
| Pm6 | 7896900 | A | G | CCTCAAAACCTCCCTCCTTC | GAGGAGGTGAAATTCCCACA | Y |
| Pm6 | 919665 | G | A | AGGCCTGCATCTGAGAATGT | ATGACTTGTGGCCTTCCAAC | Y |
| Pm6 | 5317315 | T | C | CTTTGATTTGATTCGCGCTT | AGGCCCACCATTTCTACCAT | N |
| Pm6 | 7648029 | T | C | TTGCGGTTCTTGAATGATGA | GGAGTCCGGTGAGATTGAAA | Y |
| Pm7 | 3015125 | G | A | TTTCACGGCTTTCCATATCC | GGGACTTTTGCCATGTCAGT | Y |
| Pm7 | 722218 | A | G | GGGAGGCAGATGAGATTGAA | GGTCATAAATGGGTGCCATC | Y |
| Pm7 | 8415104 | G | A | TGAGGGCTGTAGGTAGTGAGG | TCACATGCTAATGCACCGAT | Y |
| Pm7 | 16323406 | G | A | GGAAGAGGAGCGGGAATATC | GCAGGCTTCAAAGAAGTTGG | Y |
| Pm7 | 14837240 | C | T | CTGCCAGTCCTGACATGAAA | ATGGACAAAGGTGAAGACGG | Y |
| Pm7 | 12797436 | C | T | AACCATACCTCACTCGTCCG | TCAGTCTCAGCACATCCCAG | Y |
| Pm7 | 13104513 | A | C | TACCACAACTCAACACCCCA | CAGTTCTGGGACGGCTTTAG | N |
| Pm7 | 13678605 | T | G | AAACCAATCCAGGAAGACCC | AATGGAGAGGCACATTGAGG | Y |
| Pm7 | 9529134 | G | C | GTTTTGTCTGCTTCGGCTTC | TACCGATGCATTGAGCAAAA | Y |
| Pm7 | 7878252 | C | A | CATTCGATCTCTGCGCTCTT | AATTGTGAGCCTTGTCCCAG | Y |
| Pm7 | 7431325 | A | G | TTTTCAAAGCAAACGTCGTG | CCACGACGGTGAATAATTTTG | N |
| Pm7 | 11540708 | A | T | ACATCAGCTCCTGCTTTTGG | ATGTTGTTGGCTGAGCTCCT | Y |
| Pm7 | 16290605 | C | G | GACAGTCCTTGCCCTAGCAG | AGATCAACACCCGTCCACAT | Y |
| Pm7 | 567710 | C | T | CATGGCGTAGCTTGTTCAGA | TGATTTGCAGCGGATCTATG | Y |
| Pm7 | 10348726 | T | C | TTTCAGGGACAACCCTTGAC | CATTAGCATCAGCCACCAAA | Y |
| Pm7 | 9492160 | T | C | AAGGCAATGTCCTAACCACG | CAATGGCTATGTTGTGTGGC | N |
| Pm8 | 5161710 | C | A | GCAACTACCGTTCCGTCATT | TGCTCTGTCATGCTCCTTTG | Y |
| Pm8 | 6147408 | G | T | AAAGCCTACGAGCCTCATCA | GGTTTCTTGGAACGGTTTGA | Y |
| Pm8 | 10679720 | C | T | TCCAAAAATCACAGAAGGGC | CTGCACCAAAAGCAAGACAA | Y |
| Pm8 | 9353087 | G | A | ATATTGCAGGCGACATTTCC | TCTGCCTCTCTGCCTCTTTC | N |
| Pm8 | 2106261 | T | A | ACTATGGTGTCCGAACCTGG | GGATTTCTCTGCAATCTGGC | Y |
| Pm8 | 8722239 | G | C | AACGAAAATGGATGGAGCAG | AAAGGGATGGACCCAATTTC | Y |
| Pm8 | 1476704 | C | T | TCCCTTCGTTCTAGCTTCCA | GAAATCGGGTAATTCGAGCA | N |

Y: SNP show polymorphism between weeping and upright cultivars of mei. N: SNP without polymorphism between weeping and upright cultivars of mei.
